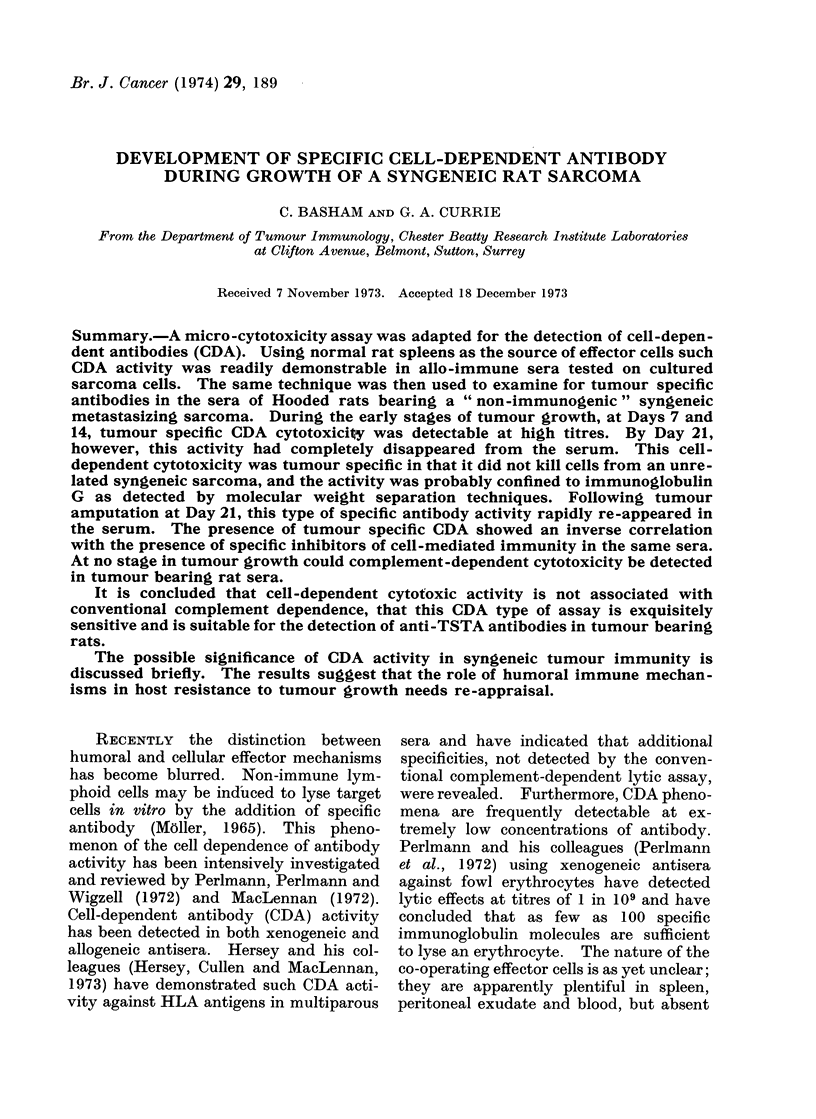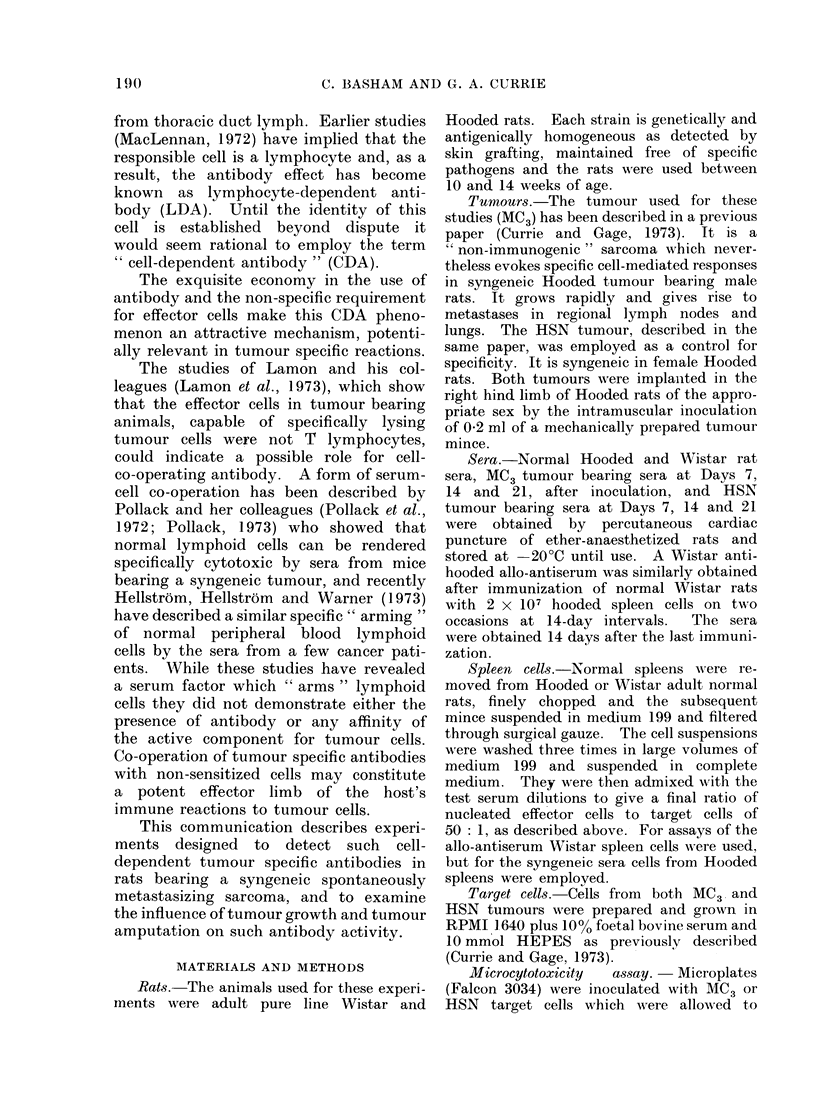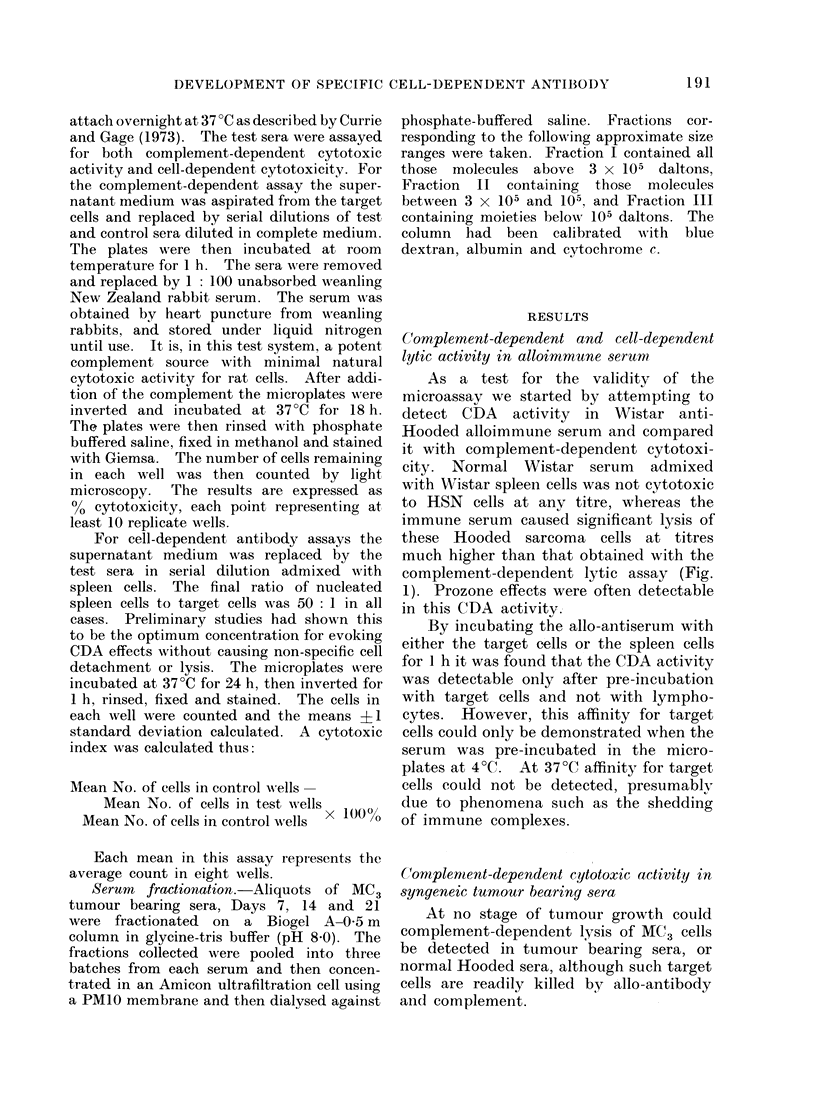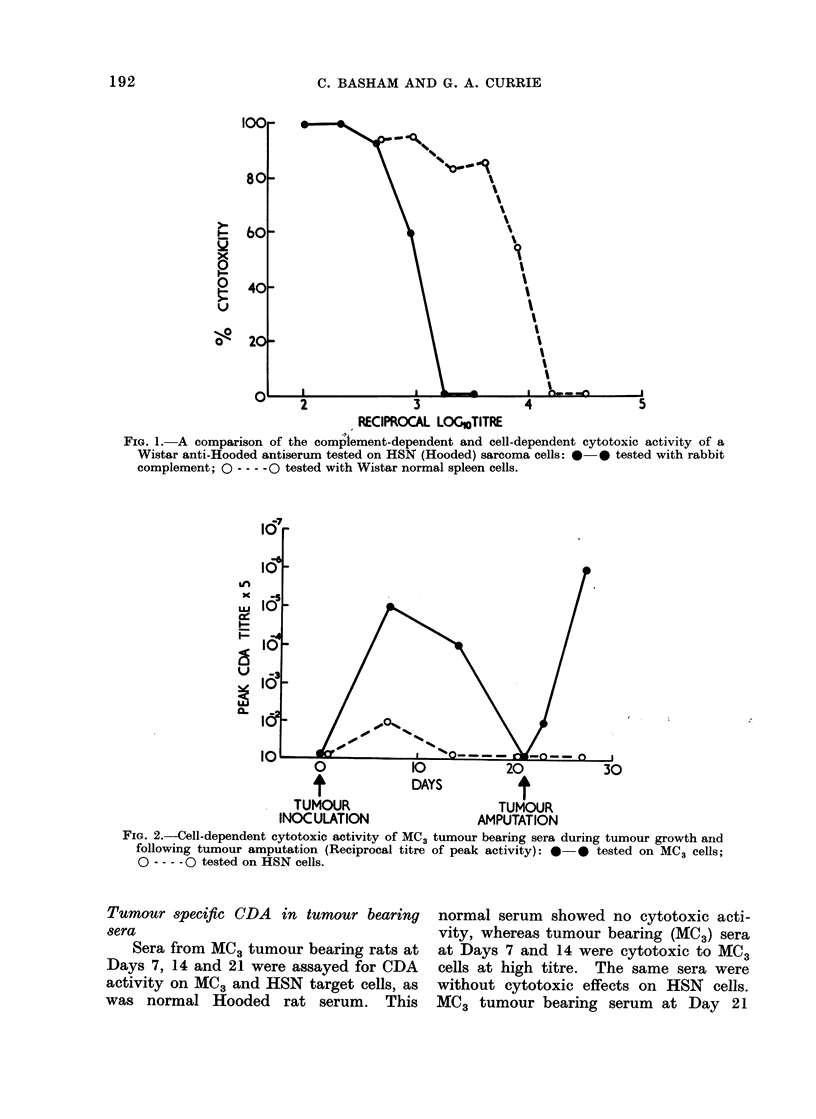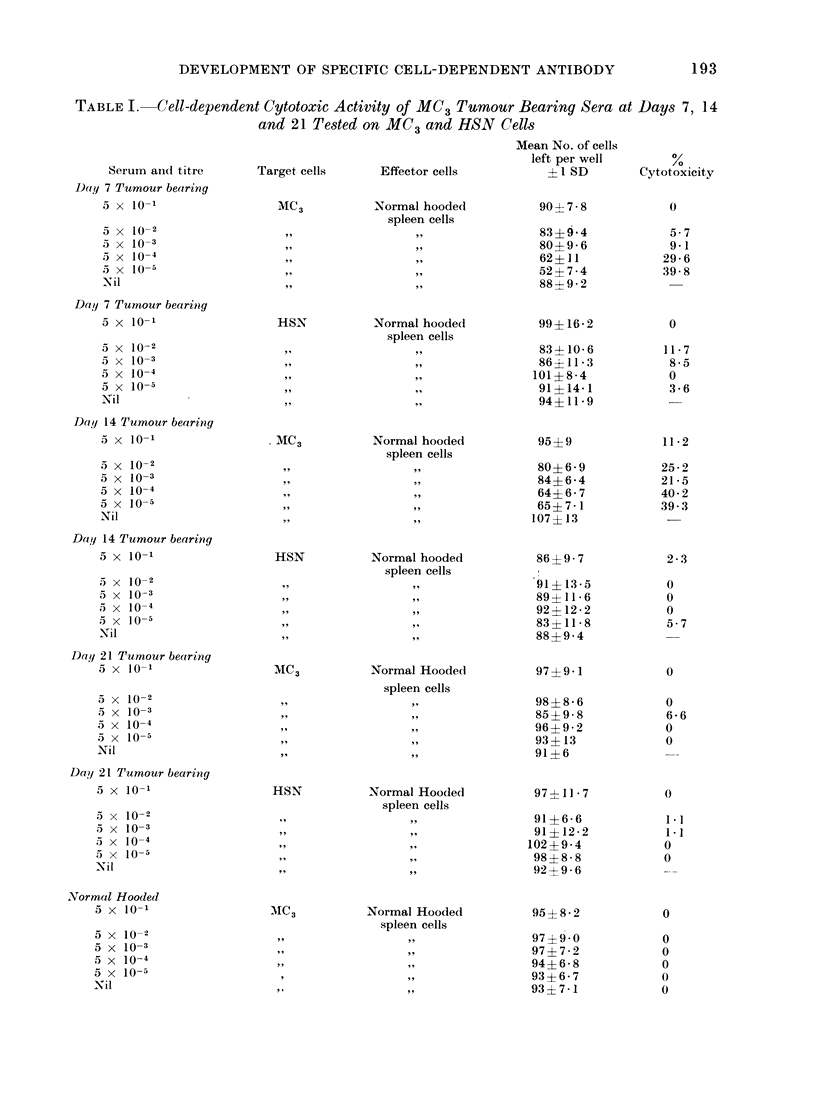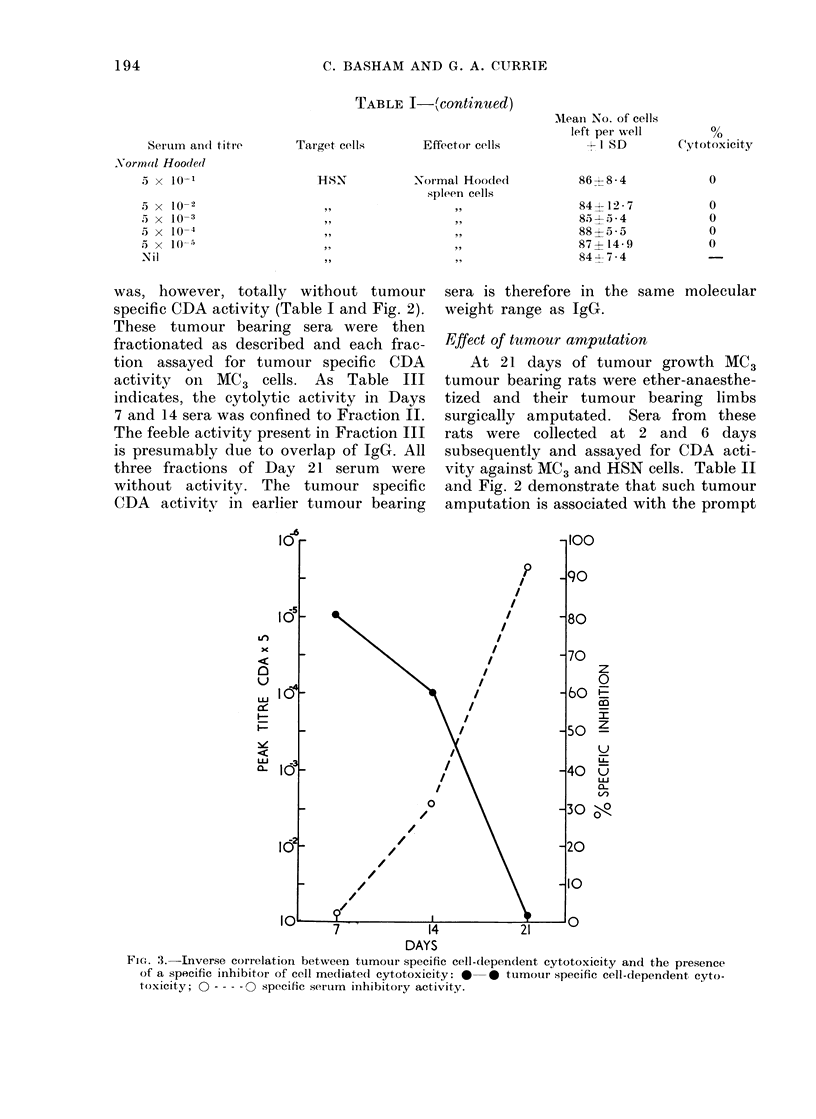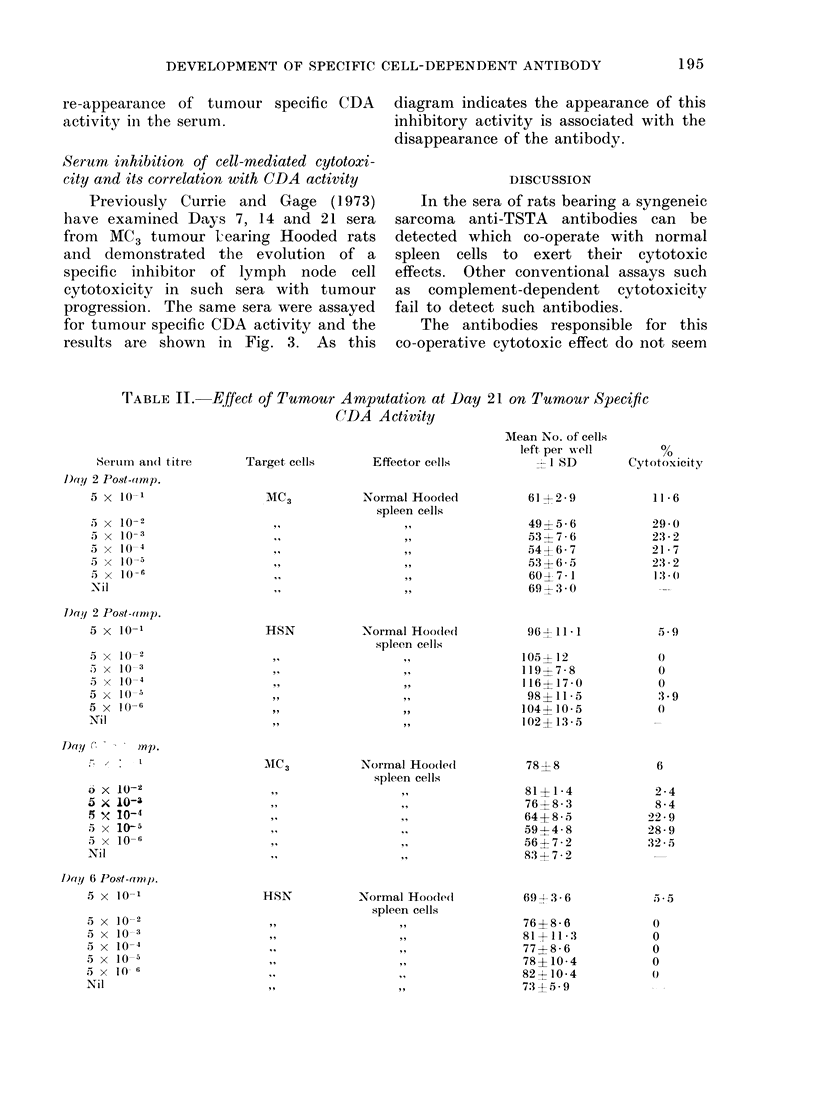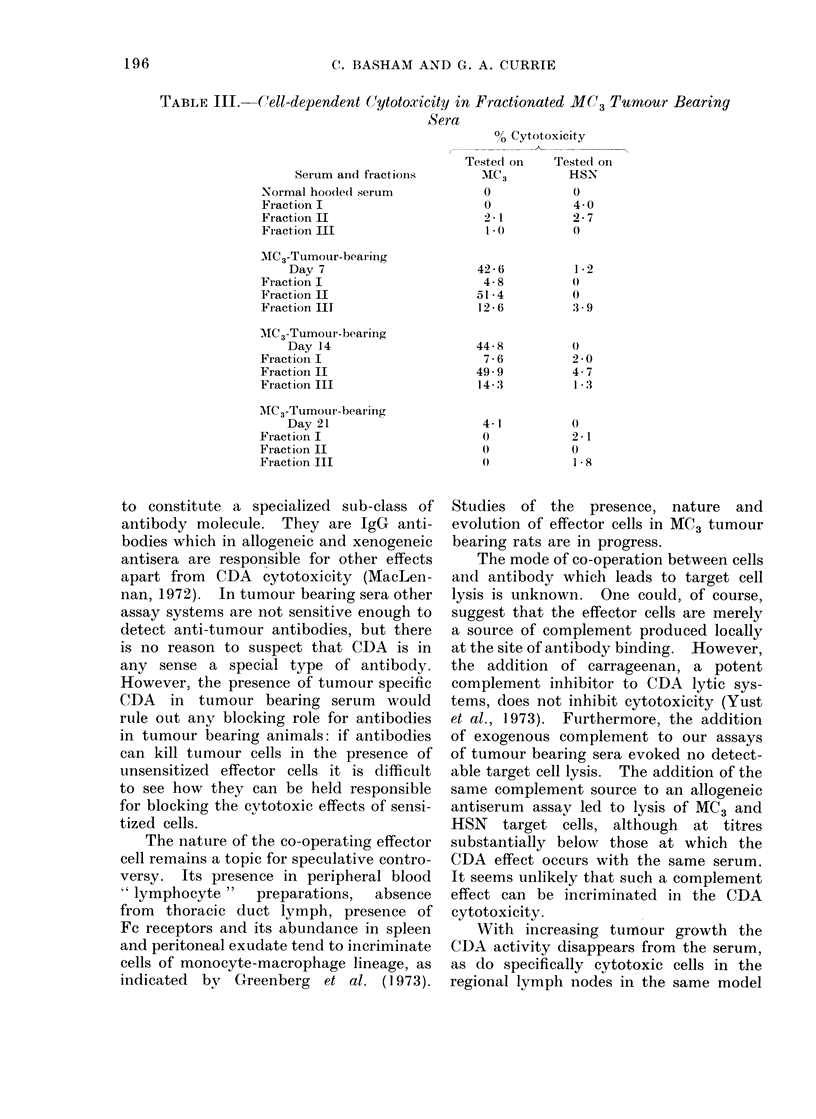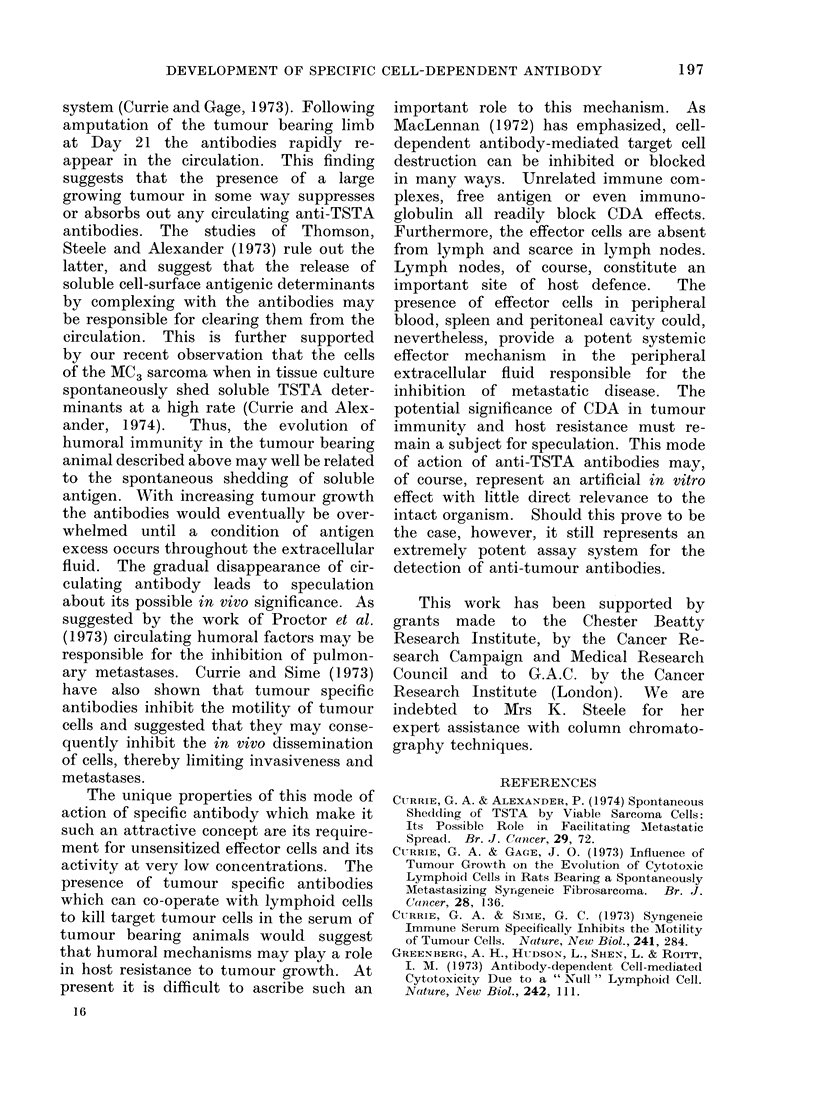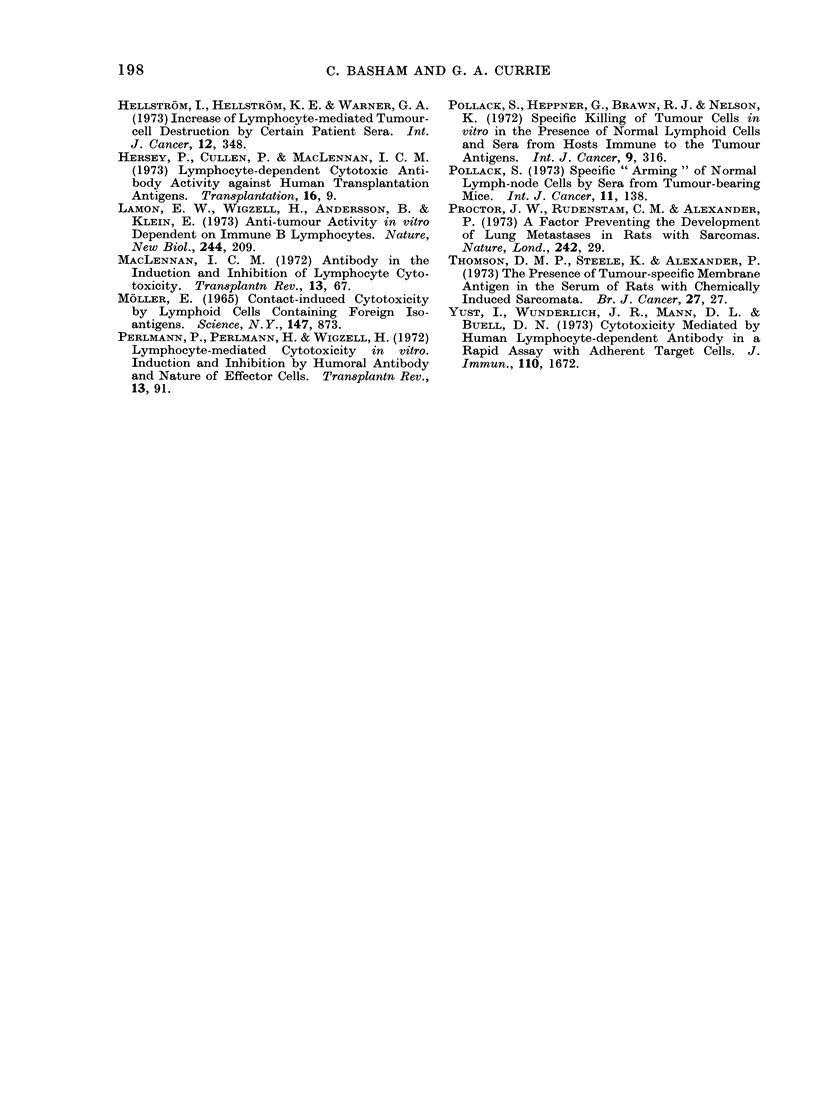# Development of Specific Cell-dependent Antibody During Growth of a Syngeneic Rat Sarcoma

**DOI:** 10.1038/bjc.1974.58

**Published:** 1974-03

**Authors:** C. Basham, G. A. Currie

## Abstract

A micro-cytotoxicity assay was adapted for the detection of cell-dependent antibodies (CDA). Using normal rat spleens as the source of effector cells such CDA activity was readily demonstrable in allo-immune sera tested on cultured sarcoma cells. The same technique was then used to examine for tumour specific antibodies in the sera of Hooded rats bearing a “non-immunogenic” syngeneic metastasizing sarcoma. During the early stages of tumour growth, at Days 7 and 14, tumour specific CDA cytotoxicity was detectable at high titres. By Day 21, however, this activity had completely disappeared from the serum. This cell-dependent cytotoxicity was tumour specific in that it did not kill cells from an unrelated syngeneic sarcoma, and the activity was probably confined to immunoglobulin G as detected by molecular weight separation techniques. Following tumour amputation at Day 21, this type of specific antibody activity rapidly re-appeared in the serum. The presence of tumour specific CDA showed an inverse correlation with the presence of specific inhibitors of cell-mediated immunity in the same sera. At no stage in tumour growth could complement-dependent cytotoxicity be detected in tumour bearing rat sera.

It is concluded that cell-dependent cytotoxic activity is not associated with conventional complement dependence, that this CDA type of assay is exquisitely sensitive and is suitable for the detection of anti-TSTA antibodies in tumour bearing rats.

The possible significance of CDA activity in syngeneic tumour immunity is discussed briefly. The results suggest that the role of humoral immune mechanisms in host resistance to tumour growth needs re-appraisal.


Br. J. Cancer (1974) 29, 189

### DEVELOPMENT OF SPECIFIC CELL-DEPENDENT ANTIBODY DURING GROWTH OF A SYNGENEIC RAT SARCOMA

C. BASHAM AND G. A. CURRIE

*From the Department of Tumour Immunology, Chester Beatty Research Institute Laboratories at Clifton Avenue, Belmont, Sutton, Surrey*

Received 7 November 1973. Accepted 18 December 1973

**Summary.—A micro-cytotoxicity assay was adapted for the detection of cell-dependent antibodies (CDA). Using normal rat spleens as the source of effector cells such CDA activity was readily demonstrable in allo-immune sera tested on cultured sarcoma cells. The same technique was then used to examine for tumour specific antibodies in the sera of Hooded rats bearing a " non-immunogenic " syngeneic metastasizing sarcoma. During the early stages of tumour growth, at Days 7 and 14, tumour specific CDA cytotoxicity was detectable at high titres. By Day 21, however, this activity had completely disappeared from the serum. This cell-dependent cytotoxicity was tumour specific in that it did not kill cells from an unrelated syngeneic sarcoma, and the activity was probably confined to immunoglobulin G as detected by molecular weight separation techniques. Following tumour amputation at Day 21, this type of specific antibody activity rapidly re-appeared in the serum. The presence of tumour specific CDA showed an inverse correlation with the presence of specific inhibitors of cell-mediated immunity in the same sera. At no stage in tumour growth could complement-dependent cytotoxicity be detected in tumour bearing rat sera.**

**It is concluded that cell-dependent cytotoxic activity is not associated with conventional complement dependence, that this CDA type of assay is exquisitely sensitive and is suitable for the detection of anti-TSTA antibodies in tumour bearing rats.**

**The possible significance of CDA activity in syngeneic tumour immunity is discussed briefly. The results suggest that the role of humoral immune mechanisms in host resistance to tumour growth needs re-appraisal.**

RECENTLY the distinction between humoral and cellular effector mechanisms has become blurred. Non-immune lymphoid cells may be induced to lyse target cells *in vitro* by the addition of specific antibody (Möller, 1965). This phenomenon of the cell dependence of antibody activity has been intensively investigated and reviewed by Perlmann, Perlmann and Wigzell (1972) and MacLennan (1972). Cell-dependent antibody (CDA) activity has been detected in both xenogeneic and allogeneic antisera. Hersey and his colleagues (Hersey, Cullen and MacLennan, 1973) have demonstrated such CDA activity against HLA antigens in multiparous sera and have indicated that additional specificities, not detected by the conventional complement-dependent lytic assay, were revealed. Furthermore, CDA phenomena are frequently detectable at extremely low concentrations of antibody. Perlmann and his colleagues (Perlmann *et al.*, 1972) using xenogeneic antisera against fowl erythrocytes have detected lytic effects at titres of 1 in $10^9$ and have concluded that as few as 100 specific immunoglobulin molecules are sufficient to lyse an erythrocyte. The nature of the co-operating effector cells is as yet unclear; they are apparently plentiful in spleen, peritoneal exudate and blood, but absent from thoracic duct lymph. Earlier studies (MacLennan, 1972) have implied that the responsible cell is a lymphocyte and, as a result, the antibody effect has become known as lymphocyte-dependent antibody (LDA). Until the identity of this cell is established beyond dispute it would seem rational to employ the term " cell-dependent antibody " (CDA).

The exquisite economy in the use of antibody and the non-specific requirement for effector cells make this CDA phenomenon an attractive mechanism, potentially relevant in tumour specific reactions.

The studies of Lamon and his colleagues (Lamon *et al.*, 1973), which show that the effector cells in tumour bearing animals, capable of specifically lysing tumour cells were not T lymphocytes, could indicate a possible role for cell-co-operating antibody. A form of serum-cell co-operation has been described by Pollack and her colleagues (Pollack *et al.*, 1972; Pollack, 1973) who showed that normal lymphoid cells can be rendered specifically cytotoxic by sera from mice bearing a syngeneic tumour, and recently Hellström, Hellström and Warner (1973) have described a similar specific " arming " of normal peripheral blood lymphoid cells by the sera from a few cancer patients. While these studies have revealed a serum factor which " arms " lymphoid cells they did not demonstrate either the presence of antibody or any affinity of the active component for tumour cells. Co-operation of tumour specific antibodies with non-sensitized cells may constitute a potent effector limb of the host's immune reactions to tumour cells.

This communication describes experiments designed to detect such cell-dependent tumour specific antibodies in rats bearing a syngeneic spontaneously metastasizing sarcoma, and to examine the influence of tumour growth and tumour amputation on such antibody activity.

### MATERIALS AND METHODS

*Rats.*—The animals used for these experiments were adult pure line Wistar and Hooded rats. Each strain is genetically and antigenically homogeneous as detected by skin grafting, maintained free of specific pathogens and the rats were used between 10 and 14 weeks of age.

*Tumours.*—The tumour used for these studies ($MC_3$) has been described in a previous paper (Currie and Gage, 1973). It is a " non-immunogenic " sarcoma which nevertheless evokes specific cell-mediated responses in syngeneic Hooded tumour bearing male rats. It grows rapidly and gives rise to metastases in regional lymph nodes and lungs. The HSN tumour, described in the same paper, was employed as a control for specificity. It is syngeneic in female Hooded rats. Both tumours were implanted in the right hind limb of Hooded rats of the appropriate sex by the intramuscular inoculation of 0·2 ml of a mechanically prepared tumour mince.

*Sera.*—Normal Hooded and Wistar rat sera, $MC_3$ tumour bearing sera at Days 7, 14 and 21, after inoculation, and HSN tumour bearing sera at Days 7, 14 and 21 were obtained by percutaneous cardiac puncture of ether-anaesthetized rats and stored at $-20°C$ until use. A Wistar anti-hooded allo-antiserum was similarly obtained after immunization of normal Wistar rats with $2 \times 10^7$ hooded spleen cells on two occasions at 14-day intervals. The sera were obtained 14 days after the last immunization.

*Spleen cells.*—Normal spleens were removed from Hooded or Wistar adult normal rats, finely chopped and the subsequent mince suspended in medium 199 and filtered through surgical gauze. The cell suspensions were washed three times in large volumes of medium 199 and suspended in complete medium. They were then admixed with the test serum dilutions to give a final ratio of nucleated effector cells to target cells of 50 : 1, as described above. For assays of the allo-antiserum Wistar spleen cells were used, but for the syngeneic sera cells from Hooded spleens were employed.

*Target cells.*—Cells from both $MC_3$ and HSN tumours were prepared and grown in RPMI 1640 plus 10% foetal bovine serum and 10 mmol HEPES as previously described (Currie and Gage, 1973).

*Microcytotoxicity assay.* — Microplates (Falcon 3034) were inoculated with $MC_3$ or HSN target cells which were allowed to attach overnight at 37 °C as described by Currie and Gage (1973). The test sera were assayed for both complement-dependent cytotoxic activity and cell-dependent cytotoxicity. For the complement-dependent assay the supernatant medium was aspirated from the target cells and replaced by serial dilutions of test and control sera diluted in complete medium. The plates were then incubated at room temperature for 1 h. The sera were removed and replaced by 1 : 100 unabsorbed weanling New Zealand rabbit serum. The serum was obtained by heart puncture from weanling rabbits, and stored under liquid nitrogen until use. It is, in this test system, a potent complement source with minimal natural cytotoxic activity for rat cells. After addition of the complement the microplates were inverted and incubated at 37°C for 18 h. The plates were then rinsed with phosphate buffered saline, fixed in methanol and stained with Giemsa. The number of cells remaining in each well was then counted by light microscopy. The results are expressed as % cytotoxicity, each point representing at least 10 replicate wells.

For cell-dependent antibody assays the supernatant medium was replaced by the test sera in serial dilution admixed with spleen cells. The final ratio of nucleated spleen cells to target cells was 50 : 1 in all cases. Preliminary studies had shown this to be the optimum concentration for evoking CDA effects without causing non-specific cell detachment or lysis. The microplates were incubated at 37°C for 24 h, then inverted for 1 h, rinsed, fixed and stained. The cells in each well were counted and the means $\pm 1$ standard deviation calculated. A cytotoxic index was calculated thus:

$$\frac{\text{Mean No. of cells in control wells} - \text{Mean No. of cells in test wells}}{\text{Mean No. of cells in control wells}} \times 100\%$$

Each mean in this assay represents the average count in eight wells.

*Serum fractionation.*—Aliquots of $MC_3$ tumour bearing sera, Days 7, 14 and 21 were fractionated on a Biogel A–0·5 m column in glycine-tris buffer (pH 8·0). The fractions collected were pooled into three batches from each serum and then concentrated in an Amicon ultrafiltration cell using a PM10 membrane and then dialysed against phosphate-buffered saline. Fractions corresponding to the following approximate size ranges were taken. Fraction I contained all those molecules above $3 \times 10^5$ daltons, Fraction II containing those molecules between $3 \times 10^5$ and $10^5$, and Fraction III containing moieties below $10^5$ daltons. The column had been calibrated with blue dextran, albumin and cytochrome *c*.

### RESULTS

### *Complement-dependent and cell-dependent lytic activity in alloimmune serum*

As a test for the validity of the microassay we started by attempting to detect CDA activity in Wistar anti-Hooded alloimmune serum and compared it with complement-dependent cytotoxicity. Normal Wistar serum admixed with Wistar spleen cells was not cytotoxic to HSN cells at any titre, whereas the immune serum caused significant lysis of these Hooded sarcoma cells at titres much higher than that obtained with the complement-dependent lytic assay (Fig. 1). Prozone effects were often detectable in this CDA activity.

By incubating the allo-antiserum with either the target cells or the spleen cells for 1 h it was found that the CDA activity was detectable only after pre-incubation with target cells and not with lymphocytes. However, this affinity for target cells could only be demonstrated when the serum was pre-incubated in the microplates at 4°C. At 37°C affinity for target cells could not be detected, presumably due to phenomena such as the shedding of immune complexes.

### *Complement-dependent cytotoxic activity in syngeneic tumour bearing sera*

At no stage of tumour growth could complement-dependent lysis of $MC_3$ cells be detected in tumour bearing sera, or normal Hooded sera, although such target cells are readily killed by allo-antibody and complement.

FIG. 1.—A comparison of the complement-dependent and cell-dependent cytotoxic activity of a Wistar anti-Hooded antiserum tested on HSN (Hooded) sarcoma cells: ●—● tested with rabbit complement; ○ - - - - ○ tested with Wistar normal spleen cells.

FIG. 2.—Cell-dependent cytotoxic activity of $MC_3$ tumour bearing sera during tumour growth and following tumour amputation (Reciprocal titre of peak activity): ●—● tested on $MC_3$ cells; ○ - - - - ○ tested on HSN cells.

*Tumour specific CDA in tumour bearing sera*

Sera from $MC_3$ tumour bearing rats at Days 7, 14 and 21 were assayed for CDA activity on $MC_3$ and HSN target cells, as was normal Hooded rat serum. This normal serum showed no cytotoxic activity, whereas tumour bearing ($MC_3$) sera at Days 7 and 14 were cytotoxic to $MC_3$ cells at high titre. The same sera were without cytotoxic effects on HSN cells. $MC_3$ tumour bearing serum at Day 21

TABLE I.—*Cell-dependent Cytotoxic Activity of $MC_3$ Tumour Bearing Sera at Days 7, 14 and 21 Tested on $MC_3$ and HSN Cells*

| Serum and titre | Target cells | Effector cells | Mean No. of cells left per well ±1 SD | % Cytotoxicity |
|---|---|---|---|---|
| *Day 7 Tumour bearing* | | | | |
| $5 \times 10^{-1}$ | $MC_3$ | Normal hooded spleen cells | 90±7·8 | 0 |
| $5 \times 10^{-2}$ | ,, | ,, | 83±9·4 | 5·7 |
| $5 \times 10^{-3}$ | ,, | ,, | 80±9·6 | 9·1 |
| $5 \times 10^{-4}$ | ,, | ,, | 62±11 | 29·6 |
| $5 \times 10^{-5}$ | ,, | ,, | 52±7·4 | 39·8 |
| Nil | ,, | ,, | 88±9·2 | — |
| *Day 7 Tumour bearing* | | | | |
| $5 \times 10^{-1}$ | HSN | Normal hooded spleen cells | 99±16·2 | 0 |
| $5 \times 10^{-2}$ | ,, | ,, | 83±10·6 | 11·7 |
| $5 \times 10^{-3}$ | ,, | ,, | 86±11·3 | 8·5 |
| $5 \times 10^{-4}$ | ,, | ,, | 101±8·4 | 0 |
| $5 \times 10^{-5}$ | ,, | ,, | 91±14·1 | 3·6 |
| Nil | ,, | ,, | 94±11·9 | — |
| *Day 14 Tumour bearing* | | | | |
| $5 \times 10^{-1}$ | $MC_3$ | Normal hooded spleen cells | 95±9 | 11·2 |
| $5 \times 10^{-2}$ | ,, | ,, | 80±6·9 | 25·2 |
| $5 \times 10^{-3}$ | ,, | ,, | 84±6·4 | 21·5 |
| $5 \times 10^{-4}$ | ,, | ,, | 64±6·7 | 40·2 |
| $5 \times 10^{-5}$ | ,, | ,, | 65±7·1 | 39·3 |
| Nil | ,, | ,, | 107±13 | — |
| *Day 14 Tumour bearing* | | | | |
| $5 \times 10^{-1}$ | HSN | Normal hooded spleen cells | 86±9·7 | 2·3 |
| $5 \times 10^{-2}$ | ,, | ,, | 91±13·5 | 0 |
| $5 \times 10^{-3}$ | ,, | ,, | 89±11·6 | 0 |
| $5 \times 10^{-4}$ | ,, | ,, | 92±12·2 | 0 |
| $5 \times 10^{-5}$ | ,, | ,, | 83±11·8 | 5·7 |
| Nil | ,, | ,, | 88±9·4 | — |
| *Day 21 Tumour bearing* | | | | |
| $5 \times 10^{-1}$ | $MC_3$ | Normal Hooded spleen cells | 97±9·1 | 0 |
| $5 \times 10^{-2}$ | ,, | ,, | 98±8·6 | 0 |
| $5 \times 10^{-3}$ | ,, | ,, | 85±9·8 | 6·6 |
| $5 \times 10^{-4}$ | ,, | ,, | 96±9·2 | 0 |
| $5 \times 10^{-5}$ | ,, | ,, | 93±13 | 0 |
| Nil | ,, | ,, | 91±6 | — |
| *Day 21 Tumour bearing* | | | | |
| $5 \times 10^{-1}$ | HSN | Normal Hooded spleen cells | 97±11·7 | 0 |
| $5 \times 10^{-2}$ | ,, | ,, | 91±6·6 | 1·1 |
| $5 \times 10^{-3}$ | ,, | ,, | 91±12·2 | 1·1 |
| $5 \times 10^{-4}$ | ,, | ,, | 102±9·4 | 0 |
| $5 \times 10^{-5}$ | ,, | ,, | 98±8·8 | 0 |
| Nil | ,, | ,, | 92±9·6 | — |
| *Normal Hooded* | | | | |
| $5 \times 10^{-1}$ | $MC_3$ | Normal Hooded spleen cells | 95±8·2 | 0 |
| $5 \times 10^{-2}$ | ,, | ,, | 97±9·0 | 0 |
| $5 \times 10^{-3}$ | ,, | ,, | 97±7·2 | 0 |
| $5 \times 10^{-4}$ | ,, | ,, | 94±6·8 | 0 |
| $5 \times 10^{-5}$ | , | ,, | 93±6·7 | 0 |
| Nil | ,, | ,, | 93±7·1 | 0 |

TABLE I—(*continued*)

| Serum and titre | Target cells | Effector cells | Mean No. of cells left per well ± 1 SD | % Cytotoxicity |
|---|---|---|---|---|
| *Normal Hooded* | | | | |
| $5 \times 10^{-1}$ | HSN | Normal Hooded spleen cells | 86±8·4 | 0 |
| $5 \times 10^{-2}$ | ,, | ,, | 84±12·7 | 0 |
| $5 \times 10^{-3}$ | ,, | ,, | 85±5·4 | 0 |
| $5 \times 10^{-4}$ | ,, | ,, | 88±5·5 | 0 |
| $5 \times 10^{-5}$ | ,, | ,, | 87±14·9 | 0 |
| Nil | ,, | ,, | 84±7·4 | — |

was, however, totally without tumour specific CDA activity (Table I and Fig. 2). These tumour bearing sera were then fractionated as described and each fraction assayed for tumour specific CDA activity on $MC_3$ cells. As Table III indicates, the cytolytic activity in Days 7 and 14 sera was confined to Fraction II. The feeble activity present in Fraction III is presumably due to overlap of IgG. All three fractions of Day 21 serum were without activity. The tumour specific CDA activity in earlier tumour bearing sera is therefore in the same molecular weight range as IgG.

*Effect of tumour amputation*

At 21 days of tumour growth $MC_3$ tumour bearing rats were ether-anaesthetized and their tumour bearing limbs surgically amputated. Sera from these rats were collected at 2 and 6 days subsequently and assayed for CDA activity against $MC_3$ and HSN cells. Table II and Fig. 2 demonstrate that such tumour amputation is associated with the prompt

FIG. 3.—Inverse correlation between tumour specific cell-dependent cytotoxicity and the presence of a specific inhibitor of cell mediated cytotoxicity: ●—● tumour specific cell-dependent cytotoxicity; ○ - - - -○ specific serum inhibitory activity.

re-appearance of tumour specific CDA activity in the serum.

### *Serum inhibition of cell-mediated cytotoxicity and its correlation with CDA activity*

Previously Currie and Gage (1973) have examined Days 7, 14 and 21 sera from $MC_3$ tumour bearing Hooded rats and demonstrated the evolution of a specific inhibitor of lymph node cell cytotoxicity in such sera with tumour progression. The same sera were assayed for tumour specific CDA activity and the results are shown in Fig. 3. As this diagram indicates the appearance of this inhibitory activity is associated with the disappearance of the antibody.

### DISCUSSION

In the sera of rats bearing a syngeneic sarcoma anti-TSTA antibodies can be detected which co-operate with normal spleen cells to exert their cytotoxic effects. Other conventional assays such as complement-dependent cytotoxicity fail to detect such antibodies.

The antibodies responsible for this co-operative cytotoxic effect do not seem

TABLE II.—*Effect of Tumour Amputation at Day 21 on Tumour Specific CDA Activity*

| Serum and titre | Target cells | Effector cells | Mean No. of cells left per well ±1 SD | % Cytotoxicity |
|---|---|---|---|---|
| *Day 2 Post-amp.* | | | | |
| $5 \times 10^{-1}$ | $MC_3$ | Normal Hooded spleen cells | $61 \pm 2 \cdot 9$ | 11·6 |
| $5 \times 10^{-2}$ | ,, | ,, | $49 \pm 5 \cdot 6$ | 29·0 |
| $5 \times 10^{-3}$ | ,, | ,, | $53 \pm 7 \cdot 6$ | 23·2 |
| $5 \times 10^{-4}$ | ,, | ,, | $54 \pm 6 \cdot 7$ | 21·7 |
| $5 \times 10^{-5}$ | ,, | ,, | $53 \pm 6 \cdot 5$ | 23·2 |
| $5 \times 10^{-6}$ | ,, | ,, | $60 \pm 7 \cdot 1$ | 13·0 |
| Nil | ,, | ,, | $69 \pm 3 \cdot 0$ | — |
| *Day 2 Post-amp.* | | | | |
| $5 \times 10^{-1}$ | HSN | Normal Hooded spleen cells | $96 \pm 11 \cdot 1$ | 5·9 |
| $5 \times 10^{-2}$ | ,, | ,, | $105 \pm 12$ | 0 |
| $5 \times 10^{-3}$ | ,, | ,, | $119 \pm 7 \cdot 8$ | 0 |
| $5 \times 10^{-4}$ | ,, | ,, | $116 \pm 17 \cdot 0$ | 0 |
| $5 \times 10^{-5}$ | ,, | ,, | $98 \pm 11 \cdot 5$ | 3·9 |
| $5 \times 10^{-6}$ | ,, | ,, | $104 \pm 10 \cdot 5$ | 0 |
| Nil | ,, | ,, | $102 \pm 13 \cdot 5$ | — |
| *Day* 6 [illegible] *mp.* | | | | |
| [illegible] | $MC_3$ | Normal Hooded spleen cells | $78 \pm 8$ | 6 |
| $5 \times 10^{-2}$ | ,, | ,, | $81 \pm 1 \cdot 4$ | 2·4 |
| $5 \times 10^{-3}$ | ,, | ,, | $76 \pm 8 \cdot 3$ | 8·4 |
| $5 \times 10^{-4}$ | ,, | ,, | $64 \pm 8 \cdot 5$ | 22·9 |
| $5 \times 10^{-5}$ | ,, | ,, | $59 \pm 4 \cdot 8$ | 28·9 |
| $5 \times 10^{-6}$ | ,, | ,, | $56 \pm 7 \cdot 2$ | 32·5 |
| Nil | ,, | ,, | $83 \pm 7 \cdot 2$ | — |
| *Day 6 Post-amp.* | | | | |
| $5 \times 10^{-1}$ | HSN | Normal Hooded spleen cells | $69 \pm 3 \cdot 6$ | 5·5 |
| $5 \times 10^{-2}$ | ,, | ,, | $76 \pm 8 \cdot 6$ | 0 |
| $5 \times 10^{-3}$ | ,, | ,, | $81 \pm 11 \cdot 3$ | 0 |
| $5 \times 10^{-4}$ | ,, | ,, | $77 \pm 8 \cdot 6$ | 0 |
| $5 \times 10^{-5}$ | ,, | ,, | $78 \pm 10 \cdot 4$ | 0 |
| $5 \times 10^{-6}$ | ,, | ,, | $82 \pm 10 \cdot 4$ | 0 |
| Nil | ,, | ,, | $73 \pm 5 \cdot 9$ | — |

TABLE III.—*Cell-dependent Cytotoxicity in Fractionated $MC_3$ Tumour Bearing Sera*

| Serum and fractions | % Cytotoxicity Tested on $MC_3$ | Tested on HSN |
|---|---|---|
| Normal hooded serum | 0 | 0 |
| Fraction I | 0 | 4·0 |
| Fraction II | 2·1 | 2·7 |
| Fraction III | 1·0 | 0 |
| $MC_3$-Tumour-bearing | | |
| Day 7 | 42·6 | 1·2 |
| Fraction I | 4·8 | 0 |
| Fraction II | 51·4 | 0 |
| Fraction III | 12·6 | 3·9 |
| $MC_3$-Tumour-bearing | | |
| Day 14 | 44·8 | 0 |
| Fraction I | 7·6 | 2·0 |
| Fraction II | 49·9 | 4·7 |
| Fraction III | 14·3 | 1·3 |
| $MC_3$-Tumour-bearing | | |
| Day 21 | 4·1 | 0 |
| Fraction I | 0 | 2·1 |
| Fraction II | 0 | 0 |
| Fraction III | 0 | 1·8 |

to constitute a specialized sub-class of antibody molecule. They are IgG antibodies which in allogeneic and xenogeneic antisera are responsible for other effects apart from CDA cytotoxicity (MacLennan, 1972). In tumour bearing sera other assay systems are not sensitive enough to detect anti-tumour antibodies, but there is no reason to suspect that CDA is in any sense a special type of antibody. However, the presence of tumour specific CDA in tumour bearing serum would rule out any blocking role for antibodies in tumour bearing animals: if antibodies can kill tumour cells in the presence of unsensitized effector cells it is difficult to see how they can be held responsible for blocking the cytotoxic effects of sensitized cells.

The nature of the co-operating effector cell remains a topic for speculative controversy. Its presence in peripheral blood "lymphocyte" preparations, absence from thoracic duct lymph, presence of Fc receptors and its abundance in spleen and peritoneal exudate tend to incriminate cells of monocyte-macrophage lineage, as indicated by Greenberg *et al.* (1973). Studies of the presence, nature and evolution of effector cells in $MC_3$ tumour bearing rats are in progress.

The mode of co-operation between cells and antibody which leads to target cell lysis is unknown. One could, of course, suggest that the effector cells are merely a source of complement produced locally at the site of antibody binding. However, the addition of carrageenan, a potent complement inhibitor to CDA lytic systems, does not inhibit cytotoxicity (Yust *et al.*, 1973). Furthermore, the addition of exogenous complement to our assays of tumour bearing sera evoked no detectable target cell lysis. The addition of the same complement source to an allogeneic antiserum assay led to lysis of $MC_3$ and HSN target cells, although at titres substantially below those at which the CDA effect occurs with the same serum. It seems unlikely that such a complement effect can be incriminated in the CDA cytotoxicity.

With increasing tumour growth the CDA activity disappears from the serum, as do specifically cytotoxic cells in the regional lymph nodes in the same model system (Currie and Gage, 1973). Following amputation of the tumour bearing limb at Day 21 the antibodies rapidly reappear in the circulation. This finding suggests that the presence of a large growing tumour in some way suppresses or absorbs out any circulating anti-TSTA antibodies. The studies of Thomson, Steele and Alexander (1973) rule out the latter, and suggest that the release of soluble cell-surface antigenic determinants by complexing with the antibodies may be responsible for clearing them from the circulation. This is further supported by our recent observation that the cells of the $MC_3$ sarcoma when in tissue culture spontaneously shed soluble TSTA determinants at a high rate (Currie and Alexander, 1974). Thus, the evolution of humoral immunity in the tumour bearing animal described above may well be related to the spontaneous shedding of soluble antigen. With increasing tumour growth the antibodies would eventually be overwhelmed until a condition of antigen excess occurs throughout the extracellular fluid. The gradual disappearance of circulating antibody leads to speculation about its possible *in vivo* significance. As suggested by the work of Proctor *et al.* (1973) circulating humoral factors may be responsible for the inhibition of pulmonary metastases. Currie and Sime (1973) have also shown that tumour specific antibodies inhibit the motility of tumour cells and suggested that they may consequently inhibit the *in vivo* dissemination of cells, thereby limiting invasiveness and metastases.

The unique properties of this mode of action of specific antibody which make it such an attractive concept are its requirement for unsensitized effector cells and its activity at very low concentrations. The presence of tumour specific antibodies which can co-operate with lymphoid cells to kill target tumour cells in the serum of tumour bearing animals would suggest that humoral mechanisms may play a role in host resistance to tumour growth. At present it is difficult to ascribe such an important role to this mechanism. As MacLennan (1972) has emphasized, cell-dependent antibody-mediated target cell destruction can be inhibited or blocked in many ways. Unrelated immune complexes, free antigen or even immunoglobulin all readily block CDA effects. Furthermore, the effector cells are absent from lymph and scarce in lymph nodes. Lymph nodes, of course, constitute an important site of host defence. The presence of effector cells in peripheral blood, spleen and peritoneal cavity could, nevertheless, provide a potent systemic effector mechanism in the peripheral extracellular fluid responsible for the inhibition of metastatic disease. The potential significance of CDA in tumour immunity and host resistance must remain a subject for speculation. This mode of action of anti-TSTA antibodies may, of course, represent an artificial *in vitro* effect with little direct relevance to the intact organism. Should this prove to be the case, however, it still represents an extremely potent assay system for the detection of anti-tumour antibodies.

This work has been supported by grants made to the Chester Beatty Research Institute, by the Cancer Research Campaign and Medical Research Council and to G.A.C. by the Cancer Research Institute (London). We are indebted to Mrs K. Steele for her expert assistance with column chromatography techniques.

REFERENCES

Currie, G. A. & Alexander, P. (1974) Spontaneous Shedding of TSTA by Viable Sarcoma Cells: Its Possible Role in Facilitating Metastatic Spread. *Br. J. Cancer*, **29**, 72.

Currie, G. A. & Gage, J. O. (1973) Influence of Tumour Growth on the Evolution of Cytotoxic Lymphoid Cells in Rats Bearing a Spontaneously Metastasizing Syngeneic Fibrosarcoma. *Br. J. Cancer*, **28**, 136.

Currie, G. A. & Sime, G. C. (1973) Syngeneic Immune Serum Specifically Inhibits the Motility of Tumour Cells. *Nature, New Biol.*, **241**, 284.

Greenberg, A. H., Hudson, L., Shen, L. & Roitt, I. M. (1973) Antibody-dependent Cell-mediated Cytotoxicity Due to a " Null " Lymphoid Cell. *Nature, New Biol.*, **242**, 111.

Hellström, I., Hellström, K. E. & Warner, G. A. (1973) Increase of Lymphocyte-mediated Tumour-cell Destruction by Certain Patient Sera. *Int. J. Cancer*, **12**, 348.

Hersey, P., Cullen, P. & MacLennan, I. C. M. (1973) Lymphocyte-dependent Cytotoxic Antibody Activity against Human Transplantation Antigens. *Transplantation*, **16**, 9.

Lamon, E. W., Wigzell, H., Andersson, B. & Klein, E. (1973) Anti-tumour Activity *in vitro* Dependent on Immune B Lymphocytes. *Nature, New Biol.*, **244**, 209.

MacLennan, I. C. M. (1972) Antibody in the Induction and Inhibition of Lymphocyte Cytotoxicity. *Transplantn Rev.*, **13**, 67.

Möller, E. (1965) Contact-induced Cytotoxicity by Lymphoid Cells Containing Foreign Isoantigens. *Science, N.Y.*, **147**, 873.

Perlmann, P., Perlmann, H. & Wigzell, H. (1972) Lymphocyte-mediated Cytotoxicity *in vitro*. Induction and Inhibition by Humoral Antibody and Nature of Effector Cells. *Transplantn Rev.*, **13**, 91.

Pollack, S., Heppner, G., Brawn, R. J. & Nelson, K. (1972) Specific Killing of Tumour Cells *in vitro* in the Presence of Normal Lymphoid Cells and Sera from Hosts Immune to the Tumour Antigens. *Int. J. Cancer*, **9**, 316.

Pollack, S. (1973) Specific " Arming " of Normal Lymph-node Cells by Sera from Tumour-bearing Mice. *Int. J. Cancer*, **11**, 138.

Proctor, J. W., Rudenstam, C. M. & Alexander, P. (1973) A Factor Preventing the Development of Lung Metastases in Rats with Sarcomas. *Nature, Lond.*, **242**, 29.

Thomson, D. M. P., Steele, K. & Alexander, P. (1973) The Presence of Tumour-specific Membrane Antigen in the Serum of Rats with Chemically Induced Sarcomata. *Br. J. Cancer*, **27**, 27.

Yust, I., Wunderlich, J. R., Mann, D. L. & Buell, D. N. (1973) Cytotoxicity Mediated by Human Lymphocyte-dependent Antibody in a Rapid Assay with Adherent Target Cells. *J. Immun.*, **110**, 1672.